# Prevalence and determinant factors of unintended pregnancy among pregnant women attending antenatal clinics of Addis Zemen hospital

**DOI:** 10.1371/journal.pone.0210206

**Published:** 2019-01-30

**Authors:** Yitayal Ayalew Goshu, Azeb Ewinetu Yitayew

**Affiliations:** Department of Midwifery, College of Health Sciences, Debre Tabor University, Debre Tabor, Ethiopia; University of Ghana, GHANA

## Abstract

**Introduction:**

Unintended pregnancy is a pregnancy which is not wanted and/or not planed at the time of conception. It has a major consequence on mothers’ and newborns’ health and its prevalence remains a major health problem in Ethiopia. This study was aimed to assess the prevalence and determinants of unintended pregnancy among pregnant women attending antenatal clinics of Addis Zemen hospital.

**Methods:**

An institutional-based cross-sectional study was employed in Addis Zemen hospital from April 01 to May 30, 2018. The sampled 398 pregnant mothers were selected by systematic random sampling. The data were collected using a-pretested structured questionnaire via face to face interview and the collected data were analyzed by using SPSS Version-20. The data were summarized with frequency and cross-tabulation. Both binary and multiple logistic regressions were used in order to identify predictor variables using odds ratio at 95% confidence interval.

**Results:**

All of 398 mothers answered the questionnaire making the response rate 100%. The prevalence of unintended pregnancy was 26.1% (CI;22.1, 30.4). Women who were multigravid (AOR; 4.7: CI; 2.3, 6.8), women who were multipara (AOR; 2.8: CI; 2.6, 9.7), and women who were from rural (AOR; 2.6: CI; 1.5, 4.6) were more likely experienced unintended pregnancy than their counterparts. Women who were Muslim (AOR; 0.79: CI; 0.6, 0.90) and women who attended secondary education (AOR; 0.58: CI; 0.42, 0.78) were less likely experienced unintended pregnancy.

**Conclusion and recommendation:**

The prevalence of unintended pregnancy is high in the study area. Educational status, parity, gravity, residence, and religion were the most important predictor variables of unintended pregnancy. Reducing the prevalence of unintended pregnancy especially in the rural area is recommended.

## Introduction

According to the world health organization, unintended pregnancy is defined as a pregnancy which is not wanted and/or not planed at the time of conception[1]. Similarly, Federation of international gynecologists and obstetricians defines unintended pregnancy is a pregnancy that is mistimed or unwanted at the time of conception[2]. This unintended pregnancy happens due to different reasons like; not using family planning, failure of contraceptive methods, lack of contraceptive methods, incidental sexual intercourse, including rape, and lack of awareness regarding family planning [3].

Unintended pregnancy has a bad consequence on the mother and newborn health. Studies indicate that women who have unintended pregnancy are more likely exposed to eclampsia, preeclampsia, postpartum hemorrhage, physical abnormality and maternal death than mothers who have intended pregnancy [4]. The most common consequence of unintended pregnancy is unsafe abortion [5]. Studies done in different parts of Ethiopia reveal that the prevalence of abortion is high. According to a study done in Harrar, the prevalence of abortion was 14.4% [6]. A national study done in Ethiopia indicates that the number of induced abortion was 620,300 in 2014 [7]. Moreover; a study done in Dessie reveals that 45% of induced abortions were unsafe abortion [8]. Despite the burden of abortion is high in Ethiopia, unmet need in the country is still a major reproductive health problem. According to Ethiopian demographic health survey 2016, unmet need in Ethiopia is as high as 22% [9].

Globally, there are 216 million maternal deaths per 100,000 live births every year [10]. Moreover; in Ethiopia, the maternal mortality rate is 412 per 100, 000 live births [9]. In Ethiopia, unsafe abortion is the second common cause of maternal mortality, which accounts for 19.7% maternal mortality [11].

Articles indicate that unintended pregnancy is one of the most critical problems in the public health system that imposes substantial financial and social costs on society. Unintended pregnancy leads to the reduction of quality of life and workforce efficiency, which indicates that unintended pregnancy affects fertility indicators [4, 5].

Even if these problems are known, the prevalence of unintended pregnancy is still high worldwide. According to WHO, every year there are around 210 million pregnancies worldwide, out of these pregnancies, 44% of pregnancies are unintended and around 59% pregnancies ended by abortion. Furthermore; most of these unintended pregnancies(65 pregnancies per 1000 women) happen in developing countries like Ethiopia [10, 12]. Different studies done in Ethiopia indicate that the prevalence of unintended pregnancy is 37.8%, 36.9%, 33.3%, 36.5% and 32.9% in Adis Ababa [13], Dilla [14], Harrar [6], Wellega [15], Debre Markos [16] respectively. However, in the best of investigators knowledge, there is no a study done in Gondar state (South and North Gondar Zone) regarding unintended pregnancy.

Unintended pregnancy is high among single women, women who have multiple sexual partners, women who have lower/no educational level, women who have low income, unemployed women, women who didn’t use family planning, multiparous mothers, young women, women who traveled long time to reach health facility, women who have more children, multigravida women, women who are not visited by health professionals, women who discussed with their husband and women who desire fewer children [17–27].

By considering these high prevalence of maternal mortality, unintended pregnancy, unmet need, abortion and high consequence of unintended pregnancy; this study was aimed to assess the prevalence and determinants of unintended pregnancy among pregnant women attending antenatal clinics of Addis Zemen hospital. The findings of this study will be helpful for policymakers in order to properly allocate resources and intervene maternal and child health problems.

## Methods

### Study setting and design

An institutional-based cross-sectional study was employed. This study was conducted in Addis Zemen primary hospital from April 01 to May 30, 2018. This hospital is found in Addis Zemen town and serves for about 298,435 total population. The hospital has adult outpatient departments, emergency outpatient department, pediatrics ward, maternity ward, medical ward, surgical ward, laboratory units, pharmacy units, maternal and child health unit, neonatal intensive-care unit, and other units. Addis Zemen is an administrative town of Libo Kemkem wereda which is located in south Gondar zone 656 kilometers away from Addis Ababa,the capital city of Ethiopia. The town has an elevation of 2, 216 meters above the sea level. The main source of income for Libo Kemkem wereda community is mixed agriculture. According to figures from the Central Statistical Agency in 2015, the estimated total population of the town is 42, 983. The town has three kebelles (the smallest unit of the woreda) and 13,515 households. The town has one primary hospital, one health center, and two private clinics.

### Study population

All pregnant women who attended antenatal clinics of Addis Zemen primary hospital during the study period were the study population.

### Sample size determination and sampling procedure

The sample size for the study was determined using a single population proportion formula n = (Zα/2)^2^P (1-P) /d^2^ with assumptions of; 37.8% the population proportion of prevalence of unintended pregnancy in Addis Ababa, 95% confidence interval, the marginal error of 5% (0.05). After adding 10% non-response rate, the final sample size found to be 398. To reach the study unit, systematic random sampling was used. To determine the sampling interval, first we reviewed six months consecutive case report and found to be 3120. Since the data collection period was two months, the average two months report was determined and found to be 1040. Finally, the sampling interval was determined by dividning two months case report to sample size and the final k^th^ value was 2.6. The first comer woman during the starting time of the data collection period was considered to be the first woman, and every second woman was asked.

### Data collection

The data were collected by two diploma degree holder midwives using an interviewer-administered structured questionnaire for two months. The questionnaire was prepared originally in English and was translated into local language, Amharic for the purpose of data collection. It was translated back to English again for consistency and accuracy by language experts. The prepared questionnaire included socio-demographic parts and reproductive characteristics.

Two weeks prior to the actual data collection time, a pretest was done among 5% of sample size out of the actual study site. Two days training regarding data collection process, the purpose of the study and ethical issues was given for data collectors and supervisors. The overall data collection process was supervised through investigators.

### Operational definition

- **Means of communication:**a woman would be considered as had means of communication if the woman had at least one means of communication (television, mobile, radio) [28].
- **Ever used family planning:** A woman would be considered as ever used family planning if she used any types of contraceptives at least for three months [28].
- **Unintended pregnancy;** a woman would be considered as having unintended pregnancy if her pregnancy was unwanted and mistimed.

### Data management and analysis

After data collection, the data were manually checked for completeness and consistency. After checking manually, the collected data were coded and entered to Epidata, software version-3.1 and finally exported to SPSS Version-20 for analysis. The data were summarized with frequency and cross-tabulation and presented using graphs and tables. Mean and standard deviation was computed for continuous variables. After the model was tested through Hosmer-Lemshow goodness of fit, binary and multivariable logistic regressions were computed to see the association between dependent and independent variables. Multi-collinearity of independent variables was checked using variance inflation factor and tolerance. In binary logistic regression variables which had aP-value of <0.25were transferred into multivariate logistic regression and finally variables which had P-value <0.05 in multivariate logistic regressions were considered as predictor variables of unintended pregnancy at 95% confidence interval.

### Ethics approval and consent to participate

The ethical clearance of this study was granted by the Institute Review Board (IRB) of Debre Tabor University. This study also supported by support letter obtained from Amhara Regional Health Bureau. Every respondent was informed about the purpose of study, voluntary basis participation and as the collected data would be kept confidential. Informed verbal consent was obtained from each respondent for those who were 18^+^ years. There were three women who were under 18 years and for those women, assent form was taken from their parents. Participants consent was documented by circling either yes or no choices for a question are you volunteer to participate in the study?”.

## Result

### Socio-demographic characteristics

A total of 398 pregnant mothers participated in the study making the response rate 100%. Almost all (99.4%) of participants were Amhara in ethnicity and most of the participants (79.1%) were Orthodox Christian in religion. Nearly one-fifth participants (21.1%) were found in the age group between 15 and 24. Most of the mothers (85.9%) had at least one type of means of communication. More than half (57.8%) of women lived in rural part and around one-third of participants had a monthly household income of 50 US dollars or less. Around one-fourth (26.1%) of respondents had no formal education and three-fourth participants (74.9%) were married (Table 1).

**Table 1:** Socio-demographic characteristic of pregnant women in Addis Zemen primary hospital, Northwestern Ethiopia, 2018 (n = 398).

Characteristics	Frequency (N)	Percent (%)
**Age**		
15–24	84	21.1
25–34	157	39.4
35–49	157	39.4
**Ethnicity**		
Amhara	396	99.4
Oromo	1	.3
Shinasha	1	.3
**Religion**		
Orthodox	315	79.1
Muslim	83	20.9
**Occupation**		
Farmer	156	39.2
Governmental employee	89	22.4
Market trade vendor	106	26.6
Housewife	47	11.8
**Educational status**		
No formal education	104	26.1
Primary school	102	25.6
Secondary school	95	23.9
College and above	97	24.4
**Monthly income**		
50 or less $	134	33.7
51–100 $	69	17.3
101–150 $	50	12.6
151–200$	70	17.6
More than 200 $	75	18.8
**Marital status**		
Married	298	74.9
Divorced	54	13.6
Widowed	34	8.5
Single	12	3
**Having means of communication**		
Yes	342	85.9
No	56	14.1
**Communication (n = 342)**		
Mobile	328	95.6
Television	215	62.9
Radio	124	36.3

**NB.** For communication, the total summation of percentage is more than 100% due to multiple answers.

### Reproductive history

Among a total of 398 mothers, 104 (26.1%) of respondents’ current pregnancy was unintended (CI;22.1, 30.4). Among those whose current pregnancy was unintended, 87 (83.7%) women’s pregnancy was mistimed whereas the rest (16.3%) women’s pregnancy was unwanted. Almost all participants (99.2%) knew the source of contraceptive methods. More than three-fourths of participants (77.2%) have freely discussed with their husbands/spouses regarding contraceptive and pregnancy. Around two-thirds of participants (67.1%) have ever used family planning (Table 2).

**Table 2:** Reproductive characteristics of women in Addis Zemen primary hospital, North-western Ethiopia, 2018 (n = 398).

Variables	Frequency (N)	Percent (%)
**Pregnancy intention**		
Intended	24	9.0
unintended	244	91.0
**Gravidity**		
1–2	81	20.4
3–4	265	66.6
≥5	52	13.0
**Parity**		
Nullipara	46	11.6
Multipara	329	82.7
Grand-multi	23	5.8
**Ever experienced abortion**		
Yes	32	8.0
No	366	92.0
**Nature of abortion (n = 32)**		
Spontaneous	15	46.9
Induced	17	53.1
**Family size**		
0–3	122	30.7
4–5	169	42.5
≥6	107	26.9
**Know about the source of contraceptives**		
Yes	395	99.2
No	3	.8
**Family planning use**		
Yes	267	67.1
No	131	32.8
**Spousal communication**		
Discussed	305	77.2
Never discussed	94	22.8
Total	422	100

### Associated factors

In order to check the association between dependent and independent variables, first 13 variables were entered intothe binary logistic regression. Then eleven variables (women’s age, educational status, monthly income, occupation, marital status, gravidity, parity, religion, residence, ever used family planning and family size) were found to be a p-value of <0.25and these variables were entered into multivariable logistic regression. Finally, in multivariable logistic regression, only five variables (women’s educational status, residence, religion, gravidity, and parity) were statically significant with unintended pregnancy (P-value <0.05). Women who were multiparous were 2.6 times more likely had an unintended pregnancy when compared to nulliparous women (AOR; 2.8: CI; 2.6, 9.7). Mothers who were from rural were 2.6 times more likely had an unintended pregnancy than mothers who were from urban (AOR; 2.6: CI; 1.5, 4.6). Participants who attended secondary education were approximately 42% less likely had anunintended pregnancy when compared to participants who had no formal education (AOR; 0.58: CI; 0.42, 0.78) (Table 3).

**Table 3:** Determinant factors of unintended pregnancy in Addis Zemen primary hospital, Northwestern Ethiopia, 2018(n = 398).

Variables	Unintended pregnancy		Crude Odd Ratio (95%CI)	Adjusted Odd Ratio (95%CI)
	Yes	No		
**Age**				
15–24	28	56	1	1
25–34	42	115	.7(.4–1.3)	.4(.3–1.5)
35–49	34	125	.6(.3-.9) **	.5(.2–1.7)
**Residence**				
Urban	48	189	1	1
Rural	56	105	1.9(1.2–2.9) **	2.6(1.5–4.6) **
**Religion**				
Orthodox	88	227	1	1
Muslim	16	67	.6(.4–1.2) *	.79(.6-.90) **
**Occupation**				
Farmer	44	112	1	1
Governmental employee	20	69	.7(.4–1.4)	.4(.3–1.4)
Market trade vendor	19	87	.6(.3–1.1) *	.3(.1–1.2)
Housewife	21	26	2.0(1.1–4.1)**	1.7(.8–3.7)
**Educational status**				
No formal education	21	83	1	1
Primary school	33	69	.4(.3-.8)**	.3(.2–2.3)
Secondary school	29	66	.4 (.2-.7)**	.58(.42-.78)**
College and above	21	76	.3 (.1-.6)**	.3(.2–1.6)
**Family Monthly income**				
50 or less $	36	98	1	1
51–100 $	21	48	1.2(.6–2.3)	1.01(.8–1.67)
101–150 $	15	35	1.2(.6–2.4)	1.12(.7–1.45)
151–200$	18	52	.9(.5–1.8)	.6(.4–1.8)
More than 200 $	14	61	.6(.-1.3)*	.4(.3–1.53)
**Marital status**				
Married	73	225	1	1
Divorced/single/widowed	31	69	1. 4(.8–2.3)*	1.2(.68–1.98)
**Ever used family planning**				
Yes	22	245	1	1
No	82	49	10.6(8.6–12.6)**	1.5(.5–2.6)
**Having means of communication**				
Yes	88	254	1	
No	16	40	1.2(.6–2.1)	
**Gravidity**				
1–2	7	74	1	1
3–4	71	194	3.9(1.7–6.8)**	3.6(2.1–5.3) **
≥5	26	26	10.6(4.1–14.7)**	4.7(2.3–6.8) **
**Parity**				
1–2	34	12	1	1
3–4	64	265	3.2(1.8–4.7) **	2.8 (2.6–9.7)**
≥5	6	17	3.6(1–6.9) **	3.2(0.8–4.5)
**Spouse communication**				
Ever discussed	85	220	1	
Never discussed	19	74	.7 (.4–1.2)	
**Family size**				
0–3	29	93	1	1
4–5	42	127	1.6(0.8–1.8)*	1.4(0.6–1.8)
≥6	33	74	1.4(.6–2.6)	1.2(.6–1.6)

* Indicates p-value<0.25

** indicates p-value<0.05

## Discussion

This study revealed that the prevalence of unintended pregnancy was 26.1% (CI;22.1, 30.4). The finding of this study is comparable to a study done in Kersa(27.9%) [22] and Gelemseo General Hospital (27.1%) [21]. This figure is also comparable to a study done in northeast Ethiopia (23.5%) [25]. However; the result of this study is lower than studies done Congo (40%) [17] and Malawi (43%) [18]. This difference may be due to the sample size difference which means the current study was carried out on small sample sizes whereas the previous Congo study was conducted on large sample size. Another explanation may be due to time variation; the use of family planning is increasing time to time and this may decrease the prevalence of unintended pregnancy. The finding of this study is also lower than studies done in Addis Ababa (37.8%) [13], Dilla (36.9%) [14],Wellega (36.5%) [15], Harar (33.3%) [6],and Debre Markos (32.9%) [16]. This difference may be due to different reasons like study design difference, time variation, socio-cultural difference, demographic differences between the current study and the previous studies. For instance, a study done in Wellega is a community-based study design which assessed all pregnant women including who had no antenatal visit. Different findings indicate that women who have intended pregnancy more utilize antenatal care than women who have unintended pregnancy [29, 30]. So the difference between the current study and previous Wellega study may be study design difference. In addition to this, the difference may be due to cultural and religious difference.

Based on multivariate logistic regression analysis family size, gravidity, parity, and educational status were found to be the determinant factors of unintended pregnancy. Multiparous women were more risky to experience an unintended pregnancy. Women who were multiparous were 2.8 times more likely to had an unintended pregnancy when compared to nulliparous women(AOR; 7.1: CI; 2.6, 9.7). The finding is in line with studies done in northeast Ethiopia [25], Kersa[22], Wellega [15]and Gelmsogeneral hospital [21] which show that women who delivered three or four children more likely experienced an unintended pregnancy. This result is also similar to a study done in Malawi as it indicated nulliparous women less likely had an unintended pregnancy. This association may be due to the fact that multiparous women may deliver every type of pregnancy intention (unintended or intended) and this may increase women’s parity.

Gravidity was another predictor variable of an unintended pregnancy. When gravidity increased, the probability of exposure to unintended pregnancy was increased. Mothers who have been pregnant for 3–4 times were 3.6 times more likely experienced an unintended pregnancy when compared to mothers who have been pregnant for 2 or less times(AOR; 3.6: CI; 2.1, 7.3). Similarly, participants who have been pregnant for 5 and more times were 4.7 times more likely reported their pregnancy was unintended than who have been pregnant for 2 or less times (AOR; 4.7: CI; 2.3, 6.8). This is in agreement with studies done in Debre Markos[16], northeast[25], and Hosana[23] as the findings indicate risk of unintended pregnancy is higher in women who have been pregnant many times. This finding is also in the agreement with a study done in Harrar [6] which shows women who have been pregnant for 2 or less times were less likely reported having an unintended pregnancy than women who have been pregnant for 5 or more times. Since gravidity is the number of pregnancy women’s gravidity may increase regardless of pregnancy intention.

This study also revealed that participants who attended secondary education were less likely at risk of having an unintended pregnancy. Respondents who attended secondary school were 42% less likely experienced an unintended pregnancy than respondents who had no formal education (AOR; 0.58: CI; 0.42, 0.78). The finding of this study is in line with studies done in Dila (14), Damote Dale (26) and north-Shewa (20). This result is also supported by two national studies done in Ethiopia (24 & 27). The association of this finding may be due to the fact that educated women may read information regarding complication of unintended pregnancy including prevention of unintended pregnancy. So, more educated women may easily get family planning methods.

In this study another most important variable which significantly associated with unintended pregnancy was religion. Muslim mothers were nearly 20% less likely to report having an unintended pregnancy as compared orthodox mothers (AOR; .79: CI; 0.60, 0.90). This finding is similar toanational study done in Ethiopia that shows Muslim mothers less likely faced an unintended pregnancy than Orthodox mothers (24). This is also in agreement with a study done in Wolaita sodo as the study reveals orthodox mothers more likely experienced an unintended pregnancy than Muslim mothers (19). This association may be due the fact that Muslim mothers take every child as a gift of the Lord and even in their religion contraceptive method is not recommnded. So, Muslim mothers don’t think that there is unintended pregnancy.

This study also found that mother’s residence as a predictor variable of unintended pregnancy. Mothers who were from rural were 2.6 times more likely had an unintended pregnancy than mothers who were from urban (AOR; 2.6: CI; 1.5, 4.6). This finding is supported by a study done in Kersa which shows that women who were from rural were 2.1 times more likely faced unintended pregnancy (22). The association of residence may be due to the fact that women who are from rural can’t easily access information regarding an unintended pregnancy and contraceptive methods. So, women who are from rural may not get easily family planning methods.

We have used a high proportion of unintended pregnancy that gives the maximum sample size and it has to be taken as a strength of the study. The other strength of the study was the probability sampling method was considered. Since this study was hospital-based, it might not be a true representative of the population. Social desirability bias mighccurred since the data were collected via face to face interview. Another limitation of this study was participants’ feelings were not studied.

## Conclusion

This finding concluded that the prevalence of unintended pregnancy is relatively low in the study area. Educational status, parity, gravity, residence, and religion were the most important predictor variables of unintended pregnancy. Reducing the prevalence of unintended pregnancy especially in the rural area is recommended. A qualitative study is highly recommended to assess mothers’ feeling regarding unintended pregnancy.

## Supporting information

S1 File Amharic version questionnaire. “Questions used to assess unintended pregnancy”.
(DOCX)

S2 File English version questionaire. “Questions used to assess unintended pregnancy”.
(DOCX)

## Abbreviations

AOR: Adjusted Odds Ratio
CI: Confidence Interval
USA: United States of America
