# Supplementary material for: Prevalence and determinant factors of unintended pregnancy among pregnant women attending antenatal clinics of Addis Zemen hospital
Source: PLoS One. 2019 Jan 30;14(1):e0210206. doi: 10.1371/journal.pone.0210206 (PMC6353093; doi:10.1371/journal.pone.0210206)
Supplement: S1 File — “Questions used to assess unintended pregnancy”. (DOCX) [file pone.0210206.s001.docx]

**በአማርኛ የተዘጋጀ መጠይቅ**

**ክፍል አንድ የማህበራዊ እና ስነህዝብ በተመለከተ የሚዳስሱ ጥያቄዎች**

| ተ.ቁ | ጥያቄ | መልስ |
| --- | --- | --- |
| 101 | ዕድሜዎት ስንት ነው? | በዓመት--------------- |
| 102 | የት ነው የሚኖሩት? | ሀ. ከተማ  ለ. ገጠር |
| 103 | የጋብቻ ሁኔታዎ ምንድን ነው? | ሀ. ያገባች  ለ. የፈታች  ሐ. የሞተባት  መ. ያላገባች  ሠ. ተለያይተው የሚኖሩ  ረ. ሳይጋቡ አበረው እሚኖሩ |
| 104 | ሀይማኖትዎ ምንድን ነው? | ሀ. ኦርቶዶክስ  ለ. ሙሰሊም  ሐ. ፕሮቴስታንት  መ. ሌላ (ይገለጥ) _____ |
| 105 | ብሄርዎ ምንድን ነው? | ሀ. አማራ  ለ. ኦሮሞ  ሐ. ትግራይ  መ. ሌላ (ይገለጥ) __________ |
| 106 | ስራዎ ምንድን ነው? | ሀ. የቤት እመቤት  ለ. የመንግስት ሰራተኛ  ሐ. ነጋዴ  መ.የቀንሰራተኛ  ሠ. ተማሪ  ረ. ሌላ (ይገለጥ)________ |
| 107 | የትምህርት ደረጃዎ ምንድን ነው? | ሀ. ማንበብ እና መፃፍ የማትችል  ለ. ማንበብ እና መፃፍ የምትችል  ሐ. አንደኛ ደረጃ (1-8)  መ. ሁለተኛ ደረጃ (9-12)  ሠ. ኮሌጅ ወይም ዩኒቨርስቲ |
| 108 | የቤቱ አማካይ ወርሀዊ ገቢ ስንት ነዉ? | ..............................በብር |
| 109 | የመገናኛ ዘዴዎች በቤትዎ ዉስጥ ይገኛሉ |  |
| 1010 | ለጥያቄ ቁጥር መልስዎ አዎ ከሆነ የትኞቹ የመገናኛ ዘዴዎች ናቸው በቤትዎ ዉስጥ ያሉት?  **(ብዙ መምረጥ ይቻላል)** | ሀ. ሬድዮለ. ቴሌቭዥን  ሐ. ሞባይል፣ስልክ መ. የለም |

**ክፍል 2 የስነ -ተዋልዶ ሁኔታን የሚዳስሱ ጥያቄዎች**

| 201 | የእርግዝናው ሁኔታ ምንድን ነው | ሀ. የታሰበ  ለ. ያልታሰበ |
| --- | --- | --- |
| 202 | ለጥያቄ ቁጥር 201 መልስ አዎ ከሆነ እርግናው ያልተፈለገ ነው ያልታቀደ | ሀ. ያልተፈለገ  ለ. ያልታቀደ |
| 203 | እስካሁን ስንት ጊዜ አርግዘሻል  ውርጃን ያካትታል | -------------- በቁጥር |
| 204 | እስካሁን ስንት ጊዜ ወልዳሻል  ከ28ኛ ሳምንት በሗላ ሙቶ የተወለደንም ሆነ በህይወት የተወለደን ያካትታል፡፡ | -------------- በቁጥር |
| 205 | ፅንስ አቋርጠሸ ታውቂያለሽ? | ሀ. አዎ  ለ. የለም |
| 206 | ለጥያቄ ቁጥር 201 መልስ አዎ አዎ ከሆነ ፈልገሽው ነው ወይስ በራሱ? | ሀ. በራሱ  ለ. ፈልጌው |
| 207 | በቤት ውስጥ ያለው የቤተሰበ ብዛት ስንት ነው? | -------------- በቁጥር |
| 208 | የእርግዝና መቆጣጠሪያዎች የት እንደሚገኙ ታውቂያለሽ? | ሀ. አዎ  ለ. የለም |
| 209 | የእርግዝና መቆጣጠሪያ ተጠቅመሽ ታውቂያለሽ? | ሀ. አዎ  ለ. የለም |
| 210 | ከባለቤትሽ/ከፍቅር ጓደኛሽ ጋር ስለ ቤተሰብ ምጣኔ እና ስል እርግዝና እቅድ በግልፅ ትወያያለሽ? | ሀ. አዎ  ለ. የለም |

**ስለትብብርዎ ከልብ እናመሰግናለን!!!!!!!!!!**
