# Supplementary material for: Prevalence and determinant factors of unintended pregnancy among pregnant women attending antenatal clinics of Addis Zemen hospital
Source: PLoS One. 2019 Jan 30;14(1):e0210206. doi: 10.1371/journal.pone.0210206 (PMC6353093; doi:10.1371/journal.pone.0210206)
Supplement: S2 File — “Questions used to assess unintended pregnancy”. (DOCX) [file pone.0210206.s002.docx]

**English version questionnaire**

**Part I: Socio -demographic factors**

| Sr. No | **Questions** | Possible answer for questions |
| --- | --- | --- |
|  | What is your age? | ................. in years |
|  | What is your residence? | A .urban  B . rural |
|  | What is your marital status? | A. Married  B. Divorced  C. Widowed  D. Single  E. Separated  F. Cohabited |
|  | What is your religion? | A. Orthodox  B. Muslim  C. Protestant  D. Others(specify) ____________ |
|  | What is your ethnicity? | A. Amhara,  B. Oromo  C. Tgray  D. Others, specify---------- |
|  | What is your occupation? | A. Farmer  B. House wife  C. Governmental Employee  D. Merchant  E. Daily Labourer  F. Student  G. Other specify....... |
|  | What is your educational Status? | A. un able to read and write  B. able to read and write  C. Primary education(1-8)  D. Secondary education(9-12)  E. College or University |
|  | What is your monthly household income? | .................in birr |
|  | Do you have any of the following means of communication? | 1. Yes 2. No |
|  | If yes for question number 109, which types of means of communication do you have?  Multiples answers are possible | A. Radio  B. TV  C. mobile  D. None |

**Part II: Questions related to reproductive history**

| 201 | What is the intention status of this pregnancy? | 1. Intended 2. unintended |
| --- | --- | --- |
| 202 | If the answer for question number 201 is unintended, is it mistimed or unwanted? | 1. unwanted 2. mistimed |
| 203 | How many times have you been pregnant? | ……………………in number |
| 204 | How many times did give a birth? It includes alive or dead baby after 28 weeks of gestation | ……………………in number |
| 205 | Have experienced abortion? | 1. Yes 2. No |
| 206 | If yes for question number 204, what was the nature of abortion? Multiples answers are possible | 1. AKeep house clean 2. Insecticide treated bed nets 3. Drainage of mosquito breeding sites 4. Spray insecticide 5. Clothing windows and doors at night 6. Take medicine 7. Others |
| 207 | What is the family size of the household? | ………………in number |
| 208 | Do you know where contraceptive methods are avail? | 1. Yes 2. No |
| 209 | Have you ever use any types of contraceptive methods? | 1. Yes 2. No |
| 210 | Do you discus freely with your husband/spouse regarding family planning and pregnancy planning? | 1. Yes 2. No |

**Thank you for your cooperation!!!!!**
